# Topical Hydroquinone for Hyperpigmentation: A Narrative Review

**DOI:** 10.7759/cureus.48840

**Published:** 2023-11-15

**Authors:** Isabella M Fabian, Evan S Sinnathamby, Chelsi J Flanagan, Anne Lindberg, Brynne Tynes, Rucha A Kelkar, Giustino Varrassi, Shahab Ahmadzadeh, Sahar Shekoohi, Alan D Kaye

**Affiliations:** 1 School of Medicine, Louisiana State University Health Sciences Center Shreveport, Shreveport, USA; 2 School of Medicine, Louisiana State University Health Sciences Center New Orleans, New Orleans, USA; 3 School of Osteopathic Medicine, University of the Incarnate Word School of Osteopathic Medicine, San Antonio, USA; 4 School of Medicine, Medical University of South Carolina, Charleston, USA; 5 Pain Medicine, Paolo Procacci Foundation, Rome, ITA; 6 Anesthesiology, Louisiana State University Health Sciences Center Shreveport, Shreveport, USA

**Keywords:** skin lightening, cosmetics, melasma, hyperpigmentation, hydroquinone

## Abstract

Topical hydroquinone (HQ) has been used for decades to treat disorders of hyperpigmentation such as melasma, post-inflammatory hyperpigmentation, and solar lentigines. Additionally, it has been used as a skin-lightening agent for cosmetic purposes. Multiple studies have shown it to be effective in treating hyperpigmentation, though it is not without side effects. Currently, HQ is not approved for over-the-counter use in the United States. Its use is also banned in other countries, including Japan, Australia, and the European Union. Hyperpigmentation disorders affect individuals worldwide. Patients with these disorders are frequently seen in medical practices. Hyperpigmentation disorders can significantly negatively impact a person's quality of life, and agents that treat hyperpigmentation can improve patient outcomes. A comprehensive literature search was performed on HQ as a treatment for skin hyperpigmentation disorders. A literature review revealed that HQ is an effective compound for treating hyperpigmentation disorders and can be combined with other therapies for enhanced results. Evidence exists to support HQ as a topical therapy for skin hyperpigmentation. HQ is not without side effects and should be prescribed by trained professionals who can educate patients on usage. HQ can be used in pharmaceutical regimens to treat hyperpigmentation disorders.

## Introduction and background

Hydroquinone (HQ) has been a standard depigmentation agent for over five decades. It is used to treat various hyperpigmentation disorders, including melasma, solar lentigines, freckles, and post-inflammatory hyperpigmentation [1]. These hyperpigmentation disorders are commonly seen in medical practices and may have a significantly negative psychosocial impact on a patient's quality of life [2-4]. HQ has the molecular formula C6H6O2. It has been regarded as a highly versatile and widely used organic compound. Aside from its pharmaceutical applications, it is also used in the photography industry and for cosmetic purposes [5]. HQ works by inhibiting tyrosinase, an enzyme involved in melanin production. Melanin is produced by melanocytes residing in the epidermis's stratum basale layer. They are the cells responsible for skin pigmentation. When applied topically, HQ may help improve skin tone and lighten hyperpigmented spots. HQ is a metabolite of benzene, and there is much interest regarding its safety and potential side effects [6]. Several studies have raised concern over the serious adverse effects, which include skin irritation, sensitization, and ochronosis, a condition characterized by the gray-blue darkening and thickening of the skin [7,8]. As a result, HQ has several restrictions for use in cosmetic products. In fact, in the United States, over-the-counter HQ products are not currently approved by the FDA [9]. However, HQ is safe and effective when used appropriately under medical supervision and within recommended concentrations. Dermatologists often prescribe HQ-based treatments for specific skin conditions and emphasize the importance of correct usage [10]. The purpose of this analysis will be to determine the efficacy of topical HQ for disorders of hyperpigmentation. Specifically, we will discuss the mechanism of action, indications for use, comparisons to other skin-lightening agents, and contraindications.

## Review

HQ mechanism of action

HQ’s main mechanism of action for treating hypopigmentation involves the inhibition of tyrosinase, the enzyme responsible for producing melanin. Tyrosinase catalyzes the conversion of the amino acid tyrosine into melanin precursors, such as dopaquinone and dopachrome. When HQ is present, tyrosinase preferentially oxidizes it over tyrosine, producing no melanin [11]. Although HQ is a poorer substrate for tyrosinase than tyrosine, it is effectively oxidized due to the generation of catalytic amounts of dopa, which acts as a cofactor for tyrosinase [11]. Furthermore, HQ inhibits the distribution of melanosome dispersion throughout the dendritic projections [12]. This can limit pigmentation uptake among keratinocytes. HQ has also been shown to affect the cytoskeleton of melanocytes directly. Melanin-producing cells incubated with HQ have dramatic changes in morphology; they become smaller and more dendritic. HQ also influences cellular microtubule formation. Cells incubated with higher amounts of HQ have less microtubule formation and more clumping of cytoskeletal structures [12]. Actin inside melanocytes is also influenced by HQ [12]. The disruption of cellular cytoskeletal structures may help explain why HQ may have cytotoxic effects at higher doses.

Some studies also show that HQ can affect melanocyte metabolism as well. In cells treated with HQ, there was significant inhibition in DNA and RNA synthesis compared to control cell lines [13]. Specifically, thymidine and uridine incorporation into DNA and RNA was inhibited in the presence of HQ, with the effect being more pronounced in melanocytes [13]. It has been hypothesized that this disruption of cellular has a greater contribution to HQ's hypopigmentation effects than its ability to inhibit tyrosinase. It has also been shown that HQ can scavenge and trap free radicals [14]. Free radicals are highly reactive molecules that can induce oxidative stress and damage several cellular components. HQ can donate electrons to free radicals [14]. This can neutralize their harmful effects and preserve melanocyte function. Thus, HQ should be used under the direction of a dermatologist or other certified medical provider. Related to its side effect profile, HQ should not be taken in excess. Long-term excessive HQ use can result in ochronosis, a bluish-black skin discoloration. Other side effects include skin irritation and redness.

HQ indications and uses

HQ is the first-line treatment for several hyperpigmentation conditions, including melasma, chloasma, freckles, age spots, and post-inflammatory hyperpigmentation caused by acne or trauma [15]. HQ is most often used at concentrations of 2-4% [16]. Studies have demonstrated that hyperpigmentation conditions respond very well to 2% HQ, and while higher concentrations are effective, they may cause adverse effects such as skin irritation [16]. Treatment areas are typically limited to the face, décolleté, and the back of the hands [17]. Melasma is one of the most common conditions used to treat HQ. Melasma is a circumscribed hypermelanosis of sun-exposed skin that presents as symmetric, hyperpigmented macules. Melasma most commonly occurs on the cheeks, upper lips, chin, and forehead [18]. The condition is much more common in women during pregnancy - this is thought to be due to increased female hormonal activity. Although the exact pathophysiology of melasma is unknown, it is hypothesized that the development of melasma is provoked by estrogen release and that estrogen receptors on melanocytes may be overstimulated, causing these melanocytes to produce more melanin [18]. HQ is structurally similar to melanin precursors and works by inhibiting the conversion of DOPA to melanin through its inhibition of tyrosinase [19]. For the treatment of melasma, HQ can be applied to the skin once daily at concentrations ranging from 2% to 5%; the effects of treatment should be evident after about 5-7 weeks of consistent use. A HQ treatment regimen should be continued for at least three months and as long as one year [20].

HQ can also be used with other medications or ingredients to increase efficacy. According to an evidence-based literature review conducted by McKesey et al. in 2020, a combination of 3% HQ applied twice daily and sunscreen applied once daily showed 96% in the appearance of melasma compared to 81% with HQ alone [21]. Solar lentigines are another condition that can be treated with HQ. Solar lentigines are darkened age spots that occur frequently in those of Asian origin [22]. Petit et al. studied the effects of 2% HQ-cyclodextrin applied once daily on solar lentigines of the forearms of thirty Asian adults for two months - the other untreated forearm on each study participant served as a control [23]. After the two-month treatment period, the control untreated solar lentigines did not demonstrate any significant changes in appearance. The treated forearms showed lightening of the solar lentigines when evaluated using corneomelametry.

In addition to its use for melasma and solar lentigines, HQ can be used as a general depigmenting agent and is often used as an active ingredient in various products used for skin bleaching [24]. HQ is currently only approved as a pharmaceutical drug, as opposed to a cosmetic product, and must be used only under dermatological supervision [24]. Skin-lightening agents such as HQ have grown to dominate the cosmetic industry, and the process of skin-lightening has become a global public health issue that can have various racial implications and adverse effects on women’s health [25]. 

A prospective comparative study was conducted to determine the efficacy of 4% HQ cream versus 0.75% kojic acid (KA) cream in the treatment of facial melasma [26]. The study allocated sixty patients diagnosed with facial epidermal melasma into group A, receiving 4% HQ cream, and group B, receiving KA cream. Side effects and clinical responses to treatment were noted at follow-up appointments on the 4th, 8th, and 12th week [26]. The response to treatment and efficacy was assessed using the melasma area severity index (MASI) score. Previous studies have stated that creams containing 2-5% concentrations of HQ are safe and efficacious when used as topical bleaching agents [18,27]. In the study by Monteiro et al. (2013), in patients who received 0.75% KA, the MASI score decreased significantly from week 0 to week 12 (P ≤ 0.001). There was a decrease in the MASI score in patients who received 4% HQ (P ≤ 0.001), and there was also a more significant change from week 0 to week 4 in the 4% HQ group than in the 0.75% KA group [26]. This showed a better effect and faster onset with HQ over KA. Another study investigated the use of HQ and KA as skin tone-lightening treatments for the skin of adult female Wistar rats [28]. The study randomly divided 80 rats into eight groups, labeled A-H, with each group assigned to be treated with varying concentrations and combinations of HQ, KA, and aloe vera, detailed in the table below (Table 1). This study showed a disruption of the stratum corneum in the samples treated with HQ alone [28]. It is speculated that this could lead to potential fragility of the skin and increased susceptibility to ultraviolet rays and other environmental hazards. The stratum corneum disruption was not seen in the samples treated with KA alone, and in the combined KA and HQ treatment, the KA appeared to ameliorate the effects of HQ mildly. Tranexamic acid (TA) is another anti-pigmentation option that has shown better results in the treatment of epidermal melasma than HQ. A study comparing the MASI scores and Melanin Index (MI) of patients at baseline, after four weeks, and after eight weeks of treatment showed a more significant decrease in average MASI scores and average MI scores of participants treated with 3% TA when compared to those treated with 4% HQ [29]. The use of thiamidol has shown improvement in mild to moderate melasma when compared to HQ. Subjects in a study comparing these topical treatments were enrolled in either the thiamidol versus untreated control group (cell A) or the thiamidol versus HQ group (cell B) [30]. Subjects in cell A had one side of their face that they treated with thiamidol, while the other side remained untreated. Subjects in cell B had one side of their face treated with thiamidol, and the other side was treated with HQ. The results showed improvement based on MASI scores for the Thiamidol-treated side of the face after twelve weeks of treatment compared to the untreated side of the face for cell A. For cell B, both the thiamidol-treated sides and the HQ-treated sides showed improvement in pigmentation relative to pretreatment, but the thiamidol-treated sides resulted in significantly lower MASI scores than the HQ-treated sides, with some patients reporting worsening of their melasma on the HQ-treated side of their face [30].

**Table 1:** Trials Comparing Hydroquinone to Other Skin-Lightening Agents HQ: Hydroquinone, KA: kojic acid, MASI: melasma area severity index, TA: tranexamic acid

Study	Treatment Groups	Results	Conclusions
Monteiro et al. [26]	4% HQ 0.75% KA and 2.5% Vitamin C	Decrease in MASI score in weeks 0-12, significant change from weeks 0-4 with 4% HQ treatment Decrease in MASI score in weeks 0-12, no significant change from weeks 0-4 with 0.75% KA treatment	The results of treatment with hydroquinone have an earlier onset than treatment with KA
Owolabi et al. [28]	Group A, control Group B, 2% HQ Group C, 2% KA Group D, 4% HQ Group E, 4% KA Group F, 2% HQ and 2% KA Group G, 4% HQ and 4% KA Group H, 4% HQ, 4% KA, and aloe vera	Groups B and D showed greater disruption of the stratum corneum Groups F, G, and H showed less disruption of the stratum corneum Groups A, C, and E did not cause observable disruption to the stratum corneum	Hydroquinone may cause greater disruption of the stratum corneum, while KA and aloe vera may ameliorate these effects
Marpaung et al. [29]	3% TA, 4% HQ	More significant decrease in MASI score and MI in 3% TA group	3% TA cream and 4% hydroquinone cream are effective in decreasing MASI score and MI
Arrowitz et al. [30]	0.2% Thiamidol-side vs. untreated side of face 0.2% Thiamidol-side vs. 2.0% HQ-side	Significant decrease in MASI score in thiamidol-treated side vs. untreated Decrease in MASI scores in both treated sides, more significant improvement in thiamidol-treated side, some worsening noted in HQ-treated sides	0.2% Thiamidol is more effective in the treatment of epidermal melasma than 2.0% hydroquinone

HQ contraindications and side effects

Topical HQ may lead to several side effects. Common short-term side effects include allergic or irritant contact dermatitis, hypopigmentation, and post-inflammatory hyperpigmentation [15,31]. The most frequent side effect with chronic use is exogenous ochronosis resulting from homogentisic acid accumulation and deposition in the skin, which presents as erythema, papulonodules, colloid milia, and symmetric blue-black and/or gray-brown hyperpigmentation in sun-exposed areas [15-32]. Although exogenous ochronosis is largely considered permanent, dermabrasion and laser treatments have successfully resolved the condition [32,33]. Trimethylaminuria, or "fish odor syndrome," is sometimes experienced by chronic HQ users, in which the body gives off a rotten fish odor as trimethylamine. This odor is secreted in sweat, urine, saliva, etc. [32]. This effect may be due to the antioxidant properties of HQ, preventing trimethylamine from oxidizing, or it may simply be an initiating element in predisposed individuals [32]. Nail hyperpigmentation has been reported as a rare side effect, which typically manifests as brown pigmentation of the nails that resolves after termination of HQ use [15,34]. Other side effects include reduced elasticity of the skin, compromised wound healing, and peripheral neuropathy. There has been some speculation regarding the carcinogenicity of HQ due to DNA damage in rodents and a few reports of squamous cell carcinoma in human users. However, humans have insufficient evidence to confirm cancer or malignancy associated with topical use [15,31,35]. Contraindications of topical HQ use include excessive sun exposure, other drugs inducing photosensitivity, and allergy or hypersensitivity to HQ. Because HQ is absorbed systemically, limiting exposure in pregnant or breastfeeding individuals is also advised [1].

## Conclusions

HQ is a topical drug frequently applied to lighten the skin. Because it is so widely used therapeutically and cosmetically, assessing its efficacy, indications, and adverse effects is important compared to alternate treatments. HQ decreases hyperpigmentation by inhibiting the enzyme tyrosinase, which converts tyrosine into melanin precursors and alters the structure and function of melanocytes. It treats many conditions, including solar lentigines, chloasma, freckles, post-inflammatory hyperpigmentation, and melasma. Several studies have shown satisfactory improvement in these disorders with HQ. However, several potential side effects include contact dermatitis, hypopigmentation, post-inflammatory hyperpigmentation, exogenous ochronosis, nail hyperpigmentation, and trimethylaminuria. Compared to other bleaching agents, HQ showed a faster onset than KA but was less effective than tranexamic Acid and thiamidol.

In conclusion, current evidence supports the use of HA for treating hyperpigmentation and skin lightening in several disorders. Unfavorable adverse effects may limit its use in some patients. Therefore, dermatologists should diligently educate patients about proper usage and, as always, consider side effects and contraindications when prescribing HQ.
